# Eating habits matter for sleep difficulties in children and adolescents: A cross-sectional study

**DOI:** 10.3389/fped.2023.1108031

**Published:** 2023-06-12

**Authors:** Yaping Zhao, Diyang Qu, Kaixin Liang, Ran Bao, Sitong Chen

**Affiliations:** ^1^ The Library Unit, Shandong Sport University, Jinan, China; ^2^ Vanke School of Public Health, Tsinghua University, Beijing, China; ^3^ School of Psychology, Shenzhen University, Shenzhen, China; ^4^ Centre for Active Living and Learning, College of Human and Social Futures, University of Newcastle, Callaghan, NSW, Australia; ^5^ Active Living Research Program, Hunter Medical Research Institute, New Lambton Heights, NSW, Australia; ^6^ Centre for Mental Health, Shenzhen University, Guangdong, China

**Keywords:** diet, sleep quality, child, adolescent, nutrition

## Abstract

**Background:**

Sleep difficulties are a common sleep-related problem among children and adolescents. However, the association between eating habits and sleep difficulties has not been extensively studied. Therefore, this study aimed to investigate the relationship between eating habits and sleep difficulties in children and adolescents.

**Methods:**

This study utilized cross-sectional data from the 2013/2014 Health Behaviour in School-aged Children survey. A total of 213,879 young adolescents provided self-reported information on their weekday and weekend breakfast consumption, fruit and vegetable intake, sweet and soft drink consumption, and sleep difficulties. Covariates such as sex, age, family affluence, physical activity, and body mass index were also assessed. Multilevel generalized linear modelling was used to analyse the association between independent and dependent. Results were reported as odds ratios (OR) with 95% confidence intervals.

**Results:**

Of all study participants, approximately 50% were girls. Regression models indicated that more frequent breakfast consumption was associated with fewer sleep difficulties (e.g., consuming breakfast on weekdays for 5 days: OR = 1.49, 95% CI: 1.45–1.54). Fruit and vegetable consumption once a week or more was also linked to fewer sleep difficulties (all OR > 1.08, ≥ 1.07). In addition, consuming fewer sweets and soft drinks was generally associated with fewer sleep difficulties.

**Conclusion:**

This study provides evidence supporting the association between healthier eating habits and reduced sleep difficulties in children and adolescents. Future research using longitudinal or experimental designs is encouraged to confirm or negate these findings. Additionally, this study offers practical guidance for nutritional counselling professionals and sleep health promotion practitioners.

## 1. Introduction

Sleeping difficulties are increasingly prevalent among adolescents, ranging from 15% to 44% (1–3), and have become a globally recognized public health issue. The short-term and long-lasting effects of sleeping difficulties on adolescents' negative developmental outcomes have been highlighted, including a higher risk of cognitive, memory, or academic deficits, increased risk of mental health problems, poorer school performance, behavior problems, and even heart disease or a higher level of all-cause mortality (4). These adverse consequences may last into adulthood; for example, previous research shows that the level of adolescents' sleep problems predicted their adulthood sleep disturbance (5). Therefore, it has become an obligation for professionals to fully identify modifiable factors linked to sleeping difficulties among children and adolescents, which are necessary for developing tailored prevention or early intervention at an early stage.

A considerable amount of knowledge on the risk factors for sleeping difficulties has been accumulated in this field. For example, previous studies have found that lifestyle factors such as consuming excessive amounts of caffeine or prolonged use of screen media in the bedroom at night, environmental factors like excessive lighting in the bedroom, psychosocial problems such as anxiety symptoms, and even medical conditions may contribute to sleeping difficulties among adolescents (4, 6–10). However, the association between eating habits and sleeping difficulties remains understudied and lacks clear answers.

Individual eating habits, such as consuming breakfast on weekdays and weekends, consuming fruits, vegetables, sweets, and soft drinks (11), are essential for living well and can have a strong impact on adolescents' biological clock, circadian rhythm, and physiological hormones like melatonin and serotonin. These factors, in turn, can either facilitate or impair their sleep quality (12–14).

A recent exploratory study investigated the relationship between breakfast consumption and subjective sleep quality among university students. The study found that students who skipped breakfast, ate late-night snacks, or replaced meals with snacks were more likely to experience poor overall sleep quality (15). However, the study's small sample size (only 498 students) and generalization bias have raised questions about its validity. Furthermore, the underlying biological and psychological mechanisms of sleep vary from childhood to adulthood due to the developmental and maturation processes (16). Therefore, a nationally representative sample of adolescents is necessary to control the risk of false-negative or false-positive findings (17).

Although a few existing studies have found a protective role of vegetable and fruit consumption in adults' short sleep and poor sleep quality (18, 19), the influence of vegetable and fruit consumption on adolescent sleep difficulties remains unclear. For instance, only a few studies have found that adolescents with shorter sleep duration report consuming fewer fruits and vegetables than those who sleep for more than 8 h per day (20). Using 8 h of average sleep duration per day as a cut-off score for comparison may limit our understanding of the association between the frequency of vegetable and fruit intake and adolescents' general sleep difficulties.

Interestingly, the findings regarding the effects of sugar and sweet consumption on adolescent sleep difficulties are mixed. For example, a study of 287 Australian children found no association between total dietary sugar consumption and sleep problems (21). However, a study of a large sample of Korean youth found a negative association between high soft drink intake and sleep duration (22). These mixed results may be due to differences in age ranges, sample sizes, and measurement methods across studies. Therefore, additional studies with more systematic measures of eating habits during specific developmental stages are necessary to better understand these findings.

To the best of our knowledge, no studies have systematically examined the possible roles of eating habits in adolescent sleep difficulties. Additionally, the optimal frequency of these eating habits for improving adolescents' sleep quality remains unclear. Investigating these associations could provide empirical evidence for the development of dietary policies, guidelines, recommendations, or school practices for adolescents. Therefore, this study aimed to investigate the associations between adolescents' eating habits (including breakfast consumption on weekdays and weekends, fruit consumption, vegetable consumption, sweets consumption, and soft drink consumption) and sleeping difficulties using a nationally representative sample from multiple countries.

## 2. Method

### 2.1. Study design

The Health Behaviour in School-aged Children (HBSC) survey is an international, school-based study conducted in collaboration with the World Health Organization across 42 countries. This cross-sectional analysis utilized data from the 2013/2014 HBSC survey. A stratified cluster sampling method was employed to identify adolescents aged 11, 13, and 15 years, with mean ages of 11.5, 13.5, and 15.5 for each respective age group. Initially, schools were randomly selected from each participating country or region. Subsequently, the number of chosen classes depended on class size. Typically, one class was selected for each grade/year; however, additional classes were included if class sizes were smaller. Self-administered questionnaires were administered at the school level to assess health and well-being, social environments, and health behaviors among children and adolescents.

### 2.2. Measures

#### 2.2.1. Independent: eating habits

The frequency of eating habits was assessed by two questions, including breakfast on weekdays and weekends, fruits, vegetables, sweets, and soft drinks. “How often do you usually have breakfast (more than a glass of milk or fruit juice)” was used to evaluate the frequency of breakfast on weekdays and weekends. Response options were never, less than once a week, two to four times a week, five to six times a week, once a day, and more than once a day. For weekdays, response options were never, only on 1 day, and on both days. The frequency of fruits, vegetables, sweets, and soft drinks was reported by adolescents in the following question: “How many times a week do you usually eat or drink fruits/vegetables/sweets (candy or chocolate)/coke or other soft drinks that contain sugar”. Response options were never, less than once a week, two to four times a week, five to six times a week, once a day, and more than once a day. For weekdays, response options were never, only on 1 day, and on both days. Eating habits were assessed by the above questions, which were based on the Food Frequency Questionnaire and showed good reliability and validity in adolescents (23).

#### 2.2.2. Dependent: sleep difficulty

Adolescents' sleep difficulty was assessed by using a single question: “How often in the past six months did you have difficulties falling asleep?” Response options were: about every day, more than once a week, about every week, about every month, and rarely or never. There has been evidence to support this single question with good reliability and validity (24).

#### 2.2.3. Covariates

The following covariates were adjusted in the statistical analysis, including sex, age (11/13/15 years), family well-off (very/quite/average/not very/not at all), body mass index (BMI), and physical activity. Adolescents were required to self-report their height and weight, and BMI then was calculated. In terms of physical activity, adolescents were required to answer the following question: “How many days did you participate in physical activity for at least 60 min per day in the past week?” Repose options were from 0 days to 7 days. These covariates were selected on the basis of their plausible associations with the outcome measures of interest (25).

### 2.3. Statistical analysis

All the statistical analysis was performed using STATA BE 17.0 (College Station, Texas, USA). Descriptive statistics were used to report sample characteristics. Multicollinearity issue was examined prior to the formal regression modelling and the results indicated no multicollinearity issues. A three-level mixed model with ordinal regression (level 3: country; level 2: school; level 1: individual; such layers were suggested according to the HBSC study protocol) was established to assess the associations of eating habits with sleeping difficulties (treated as ordinal variable), while controlling for sex, age category, family well-off, BMI and physical activity. *meglm* command was utilized to achieve to establish the above models. Results from the model were mainly presented as the odd ratio (OR) with a 95% confidence interval (CI), as an ordinal logistic regression model was used. A prior *p*-value below 0.05 was used to ascertain statistical significance.

## 3. Results

Sample characteristics are shown in Table 1. Of the 213,879 study participants aged between 11 and 15 years, 50.8% of them were girls, and the mean of BMI was 19.5. The percentage of adolescents aged 11, 13, and 15 years was 32.2%, 34.6%, and 33.2%. In total, 39.0% of the adolescents reported their family well-off as “Average”, 34.4% self-rated as “Quite”, 19.5% rated it as “Very”, 5.7% rated as “Not very”, and 1.5% rated as “Not at all”. On average, participants reported having about 4.2 days a week of physical activity.

**Table 1.** Sample characteristics and information on covariates.

**Categorical variables**	**Proportion**
**Sex**	** **
Boy	49.2%
Girl	50.8%
**Age (years)**	** **
11	32.2%
13	34.6%
15	33.2%
**Family well-off**	** **
Very	19.5%
Quite	34.4%
Average	39.0%
Not very	5.7%
Not at all	1.5%
**Continuous variable**	**Mean**
**Physical activity days**	4.2
**Body mass index**	19.5

In Table 2, of the total sample, 63.3% of them reported eating breakfast for 5 days, and 15.7% never ate breakfast on weekdays. On weekends, the percentage of eating breakfast for 2 days was 79.5%, and only 7.1% of them never ate breakfast. In the total sample, 20.2% of adolescents ate fruit more than once daily, 15.4% of them reported for 5–6 days a week, 28.2% was 2–4 days a week, 9.5% was once a week, and 6.0% was less than once a week. Regarding eating vegetables, 16.6% and 19.0% of adolescents reported “more than once daily” and “once daily”, most of them ate vegetables less than once daily. Besides, the percentage of adolescents who reported eating sweets more than once daily was 11.7%, 5–6 days a week was 12.8%, 2–4 days a week was 28.2%, and once a week or less was 35.3%. There were 10.4% and 7.4% of adolescents who had soft drinks more than once daily and once daily, and more than half of adolescents reported once a week or less. In terms of sleep difficulty, 10.8% of participants reported having sleep difficulties every day, while 51.7% of them reported rarely or never with regard to sleep difficulties.

**Table 2.** Descriptives on independent and dependent in this study.

**Variables**	**Proportion**
**Breakfast weekdays**	** **
Never	15.7%
One day	4.0%
Two days	4.9%
Three days	6.6%
Four days	5.5%
Five days	63.3%
**Breakfast weekends**	** **
Never	7.1%
One day	13.3%
Both days	79.5%
**Eat fruit**	** **
Never	3.0%
Less than once a week	6.0%
Once a week	9.5%
2-4 days a week	28.2%
5-6 days a week	15.4%
Once daily	17.7%
More than once daily	20.2%
**Eat vegetables**	** **
Never	4.5%
Less than once a week	5.8%
Once a week	9.8%
2-4 days a week	25.2%
5-6 days a week	19.1%
Once daily	19.0%
More than once daily	16.6%
**Eat sweets**	** **
Never	4.1%
Less than once a week	12.0%
Once a week	19.2%
2-4 days a week	28.2%
5-6 days a week	12.8%
Once daily	12.1%
More than once daily	11.7%
**Consumption of soft drinks**	** **
Never	11.1%
Less than once a week	22.0%
Once a week	18.8%
2-4 days a week	21.7%
5-6 days a week	8.7%
Once daily	7.4%
More than once daily	10.4%
**Sleep difficulty**	** **
About every day	10.8%
More than once/week	10.4%
About every week	11.0%
About every month	16.1%
Rarely or never	51.7%

The results on the associations between independents and outcomes are shown in Table 3. Among the sample, those who ate breakfast on weekdays for two (OR = 1.09, CI = 1.03–1.16, *p* = 0.002), three (OR = 1.12, CI = 1.07–1.18, *p* < 0.001), four (OR = 1.18, CI = 1.12–1.25, *p* < 0.001) and 5 days (OR = 1.49, CI = 1.45–1.54, *p* < 0.001) were less likely to report sleep difficulties compared to those who did not eat breakfast for any day. Similarly, eating breakfast on weekends for one (OR = 1.16, CI = 1.10–1.22, *p* < 0.001) and 2 days (OR = 1.49, CI = 1.43–1.56, *p* < 0.001) were also negatively associated with sleep difficulties. In terms of fruits consumption, compared with not eating fruits, children who ate fruits once a week were at 1.12 times lower likelihood (CI = 1.03–1.22, *p* = 0.008), for 2–4 days a week were at 1.13 times lower likelihood (CI = 1.04–1.22, *p* = 0.003), for 5–6 days a week were at 1.11 times lower likelihood (CI = 1.03–1.21, *p* = 0.010), for once daily were at 1.15 times lower likelihood (CI = 1.06–1.25, *p* = 0.001), and for more than once daily were at 1.13 times lower likelihood (CI = 1.04–1.23, *p* = 0.003) of reporting sleep difficulties. For vegetable consumption, children who reported a higher frequency of eating vegetables were more likely to have a low level of sleep difficulties, such as once a week (OR = 1.08, CI = 1.01–1.16, *p* = 0.027), for 2–4 days a week (OR = 1.09, CI = 1.03–1.17, *p* = 0.006), for 5–6 days a week (OR = 1.07, CI = 1.00–1.15, *p* = 0.042), for once daily (OR = 1.09, CI = 1.02–1.17, *p* = 0.012) and for more than once daily (OR = 1.09, CI = 1.01–1.16, *p* = 0.021). However, compared with more than once daily sweets and soft drinks, fewer consumption of them were more likely associated with fewer sleep difficulties (except for 5–6 days). Pseudo *R*^2^ of the regression model was 0.012. More details can be found in Table 1.

**Table 3:** Results on the association between eating habits and sleep difficulties.

Dependent: sleep difficulties	OR	95% CI		*p*
Breakfast on weekday (ref = never)				
One day	1.05	0.98	1.11	0.151
Two days	1.09	1.03	1.16	0.002
Three days	1.12	1.07	1.18	<0.001
Four days	1.18	1.12	1.25	<0.001
Five days	1.49	1.45	1.54	<0.001
Breakfast on weekend (ref = never)				
One day	1.16	1.10	1.22	<0.001
Both days	1.49	1.43	1.56	<0.001
Fruits (ref = never)				
Less than once a week	1.04	0.95	1.14	0.362
Once a week	1.12	1.03	1.22	0.008
2–4 days a week	1.13	1.04	1.22	0.003
5–6 days a week	1.11	1.03	1.21	0.010
Once daily	1.15	1.06	1.25	0.001
More than once daily	1.13	1.04	1.23	0.003
Vegetables (ref = never)				
Less than once a week	1.02	0.94	1.10	0.620
Once a week	1.08	1.01	1.16	0.027
2–4 days a week	1.09	1.03	1.17	0.006
5–6 days a week	1.07	1.00	1.15	0.042
Once daily	1.09	1.02	1.17	0.012
More than once daily	1.09	1.01	1.16	0.021
Sweets (ref = more than once daily)				
Never	1.24	1.15	1.33	<0.001
Less than once a week	1.31	1.25	1.38	<0.001
Once a week	1.30	1.24	1.36	<0.001
2–4 days a week	1.24	1.19	1.30	<0.001
5–6 days a week	1.11	1.06	1.16	<0.001
Once daily	1.17	1.12	1.22	<0.001
Soft drinks (ref = more than once daily)				
Never	1.18	1.12	1.24	<0.001
Less than once a week	1.20	1.15	1.26	<0.001
Once a week	1.19	1.14	1.25	<0.001
2–4 days a week	1.14	1.09	1.20	<0.001
5–6 days a week	1.04	0.98	1.09	0.171
Once daily	1.11	1.05	1.17	<0.001

OR, odds ratio; CI, confidence interval.

## 4. Discussion

This study aims to investigate the association between eating habits and sleep difficulties in children and adolescents using a large sample based on data from the 2013/2014 round of the HBSC survey. Several interesting findings were observed. Firstly, this study found that a high frequency of breakfast consumption is associated with fewer sleep difficulties. Similarly, frequent consumption of vegetables and fruits is significantly associated with fewer sleep difficulties. Conversely, the study found that consuming more sweets and soft drinks (except for 5–6 days per week) is associated with more sleep difficulties. These findings contribute to our understanding of the relationships between eating habits and sleep health in children and adolescents. A more detailed analysis is presented below.

To the best of our knowledge, there is limited evidence regarding the associations between eating habits, particularly specific food types, and sleep difficulties among children and adolescents (26, 27). However, sleep difficulties may be regarded as clinical symptoms of overall sleep quality (25, 28). Therefore, evidence regarding eating habits and sleep quality could be useful for interpreting our research findings. Some previous studies have summarized the preliminary evidence concerning the association between eating habits and sleep quality, indicating that unhealthy eating habits were related to poor sleep quality (26, 28–30). Additionally, studies focused on workers have found that poor eating habits have a significant impact on their sleep quality (31), which is supported by later studies (32, 33). However, sleep patterns differ and change from childhood to adulthood based on various factors. Therefore, focusing on specific age-related populations is necessary. In other words, this study extends the knowledge on the relationships between healthier eating habits and better sleep quality in children and adolescents. We found that better eating habits (such as not skipping breakfast, consuming more fruits and vegetables, and limiting the intake of sweets and soft drinks) were associated with better sleep quality (i.e., fewer sleep difficulties in this study). It is worth noting that previous studies have mainly focused on the comprehensive assessment of sleep quality (28, 29, 34–36), such as using the Pittsburgh Sleep Quality Index. In this study, a single question item was used to collect children and adolescents' self-perceptions of their own sleep quality. The heterogeneity of measurements may limit our understanding of the differences between the findings of this study and other studies, which requires further verification.

Maintaining regular breakfast consumption is of great health significance for children and adolescents, as it promotes mental health (37, 38) and prevents obesity (39, 40). The results of this study indicate that a higher frequency of breakfast consumption is positively associated with better sleep quality, such as fewer sleep difficulties, which is consistent with previous studies conducted on Japanese adolescents (41). Therefore, encouraging children and adolescents to develop regular breakfast eating habits can be beneficial for promoting better sleep quality. A possible explanation for this finding is that regular breakfast consumption is associated with better mental health status, which may contribute to better sleep quality (37, 38, 41).

Synthesized evidence suggests that consuming fruits and vegetables is associated with better sleep quality (35). For example, experimental studies have shown that consuming two kiwifruits per day can significantly improve sleep quality (42). Similarly, cherries have been found to promote better sleep quality (43). In terms of vegetables, a study conducted on Korean adolescents found that consuming more vegetables was associated with better sleep quality, including fewer sleep difficulty symptoms (22), which is supported by similar studies (44, 45). Although evidence on children and adolescents is limited, this collective evidence supports our research statement that consuming more fruits and vegetables is associated with fewer sleep difficulties. Calorie restriction may be a possible mechanism for the association between eating habits and sleep difficulties, as it has been shown to promote better sleep quality (26, 29). Experimental evidence has shown that calorie restriction can be achieved through consuming most fruits and vegetables. Another possible explanation is sleep duration, which is co-dependent with better sleep quality. Appropriate sleep duration is associated with eating habits (28, 34), and healthier eating habits may be associated with better sleep quality based on appropriate sleep duration (29). Additionally, fruits and vegetables are rich in fiber, vitamins, and minerals, and high intake of these nutrients has been linked to better sleep quality (34, 44, 46).

In terms of unhealthy food items, the current study found that consuming more sweets and soft drinks was associated with more sleep difficulties, indicating poorer sleep quality. This finding is supported by some previous survey studies. For example, data from the Korean Youth Risk Behavior Surveillance indicated that poor sleep quality may be associated with higher consumption of sugar-sweetened beverages (22), which supports our finding that increased sweets consumption is related to more sleep difficulties. Many studies have confirmed the negative association between soft drink consumption and sleep quality outcomes (47–50). These previous findings partly corroborate the research findings of our study. One possible explanation for this association involves sugar, as higher sugar intake has been associated with worse sleep quality (51).

Based on the above evidence and analysis, it is reasonable to conclude that children and adolescents with healthier eating habits are more likely to experience better sleep quality. However, it is important to consider some limitations of this study. The adjusted *R*^2^ of the model is relatively small, explaining only 1.2% of the variability in sleep difficulty symptoms among young adolescents. Therefore, the findings should be interpreted with caution. Nonetheless, the model can identify potential modifiable factors that are linked to sleep difficulties among young adolescents, providing guidance for developing tailored prevention or early intervention strategies to improve sleep quality. Additionally, the cross-sectional nature of the study means that causality and directionality cannot be established from the research findings. Another limitation is that data on nutritional or food compositions, such as protein intake and carbohydrate intake, as well as some other confounding factors (e.g., developmental disorders), could not be collected due to the measurement protocol. This limitation prevents us from gaining insights into the mechanisms underlying the association between eating habits and sleep difficulties. Furthermore, the current data collected cannot reflect the dietary patterns of the study participants, inhibiting our understanding of the relationship between overall daily diet and sleep quality. It is also important to consider potential overlap between sweets and soft drinks that contain caffeine (53), such as chocolate and cola, and the effects of caffeine on sleep should be studied. Addressing these limitations is promising for generating better evidence for nutritional clinical practice and counselling.

The present study contributes to our understanding of healthy eating guidelines by providing evidence supporting the importance of increasing the frequency of breakfast consumption, as well as the intake of vegetables and fruits, among children and adolescents. Furthermore, the study highlights the need to limit sweets and soft drink consumption to no more than 5–6 days per week due to their negative association with sleep quality.

## 5. Conclusion

In summary, this study provides evidence on the relationships between dietary habits and sleep challenges in children and adolescents, highlighting the importance of breakfast, fruit, and vegetable consumption, as well as the negative impact of sweets and soft drinks. Prioritizing certain food items from a nutritional improvement standpoint could be highly beneficial in addressing sleep issues. However, due to the limitations of this study, further evidence from cohort and intervention studies is needed to strengthen our understanding of these associations.

## Data Availability

Publicly available datasets were analyzed in this study. This data can be found here: https://www.who.int/europe/initiatives/health-behaviour-in-school-aged-children-(hbsc)-study.
